# Fungal Adaptation to the Advanced Stages of Wood Decomposition: Insights from the *Steccherinum ochraceum*

**DOI:** 10.3390/microorganisms7110527

**Published:** 2019-11-05

**Authors:** Konstantin V. Moiseenko, Olga A. Glazunova, Natalia V. Shakhova, Olga S. Savinova, Daria V. Vasina, Tatiana V. Tyazhelova, Nadezhda V. Psurtseva, Tatiana V. Fedorova

**Affiliations:** 1 A. N. Bach Institute of Biochemistry, Research Center of Biotechnology, Russian Academy of Sciences, Leninsky Ave. 33/2, Moscow 119071, Russia; olga.a.glas@gmail.com (O.A.G.); savinova_os@rambler.ru (O.S.S.); d.v.vasina@gmail.com (D.V.V.); 2 Komarov Botanical Institute of the Russian Academy of Sciences, Professor Popov St. 2, St. Petersburg 197376, Russia; shakhova-11@yandex.ru; 3 N. I. Vavilov Institute of General Genetics, Russian Academy of Sciences, Moscow 119071, Russia; t.tyazhelova@gmail.com

**Keywords:** *Steccherinum ochraceum*, genome, wood decay, CAZymes, laccases, class II peroxidases, phylogeny, evolution, exoproteome

## Abstract

*Steccherinum ochraceum* is a white rot basidiomycete with wide ecological amplitude. It occurs in different regions of Russia and throughout the world, occupying different climatic zones. *S. ochraceum* colonizes stumps, trunks, and branches of various deciduous (seldom coniferous) trees. As a secondary colonizing fungus, *S. ochraceum* is mainly observed at the late decay stages. Here, we present the *de novo* assembly and annotation of the genome of *S. ochraceum*, LE-BIN 3174. This is the 8th published genome of fungus from the residual polyporoid clade and the first from the *Steccherinaceae* family. The obtained genome provides a first glimpse into the genetic and enzymatic mechanisms governing adaptation of *S. ochraceum* to an ecological niche of pre-degraded wood. It is proposed that increased number of carbohydrate-active enzymes (CAZymes) belonging to the AA superfamily and decreased number of CAZymes belonging to the GH superfamily reflects substrate preferences of *S. ochraceum*. This proposition is further substantiated by the results of the biochemical plate tests and exoproteomic study, which demonstrates that *S. ochraceum* assumes the intermediate position between typical primary colonizing fungi and litter decomposers or humus saprotrophs. Phylogenetic analysis of *S. ochraceum* laccase and class II peroxidase genes revealed the distinct evolutional origin of these genes in the *Steccherinaceae* family.

## 1. Introduction

Forest ecosystems, a land mass covered in trees, are major ecological units existing on our planet. These ecosystems are mainly responsible for such central processes in the biosphere as photosynthesis—the use of light energy to drive fixation of carbon dioxide. The largest part of the fixed carbon is utilized by trees to form a wood—the porous and fibrous structural tissue comprised of heavily lignified cells. Consequently, upon the death of a tree, wood becomes an enormous pool of organic matter (*lignum*) [1,2]. However, mainly due to the presence of lignin, wood is a highly recalcitrant structure, decomposition of which is a complex process involving several stages to release stored carbon back to the environment [3]. Besides *lignum*, many other remnants (e.g., leaves, needles, dead plant roots, and root exudates) are formed during the life cycle of a tree [4]. Being very diverse in nature and including both relatively fresh (*folia dejecta*), well-degraded (*stramentum*), and humified (*humus*) organic matter, which correspondingly form L/Oi-, F/Oe-, and H/Oa-horizons of the forest floor (O-horizon), these remnants are segregated under the term soil organic matter (SOM) [5,6].

Saprophytic basidiomycete fungi are an essential part of every forest ecosystem. Participating in every stage of wood degradation and SOM decomposition, these fungi play fundamental roles in carbon balance, soil formation, and forest regeneration [7,8,9]. While the majority of basidiomycete fungi that degrade wood cell walls belong to the order *Polyporales*, basidiomycetes that participate in the decomposition of SOM can be found in both *Polyporales* and *Agaricales* orders [4].

Many investigations performed over the past several decades clearly demonstrate that decomposer fungal communities participating in different stages of wood degradation and SOM decomposition are qualitatively different in both species composition and overall biochemical processes [10]. In general terms, the sequence of wood decomposition from standing tree to complete decay can be described as follows [11]: (1) upon the death of a tree or any of its parts, the reduced water content stimulates growth of the fungi that were latently present within a functional wood (the primary colonizing (pioneer) fungi); (2) with the advancement of wood degradation, primary colonizers are successively replaced by the secondary colonizers, possessing either higher combative ability or greater adaptation to the newly forming physicochemical conditions in pre-degraded wood; (3) as a result of structural instability, well-degraded wood fall down under the gravitational force and become in contact with the forest floor; (4) on the forest floor, degraded wood can be colonized by the fungi, whose typical substrate is SOM; at this stage a considerable overlap between wood degrading and SOM decomposing fungal communities occurs; (5) the increasing destruction and immersion of the woody material into the soil promote humification processes, and the prevalent type of fungi observed at this stage are humus saprotrophs.

Given the different physicochemical properties of the substrate occurring at the different stages of wood degradation and SOM decomposition, it is not surprising that fungi operating at these stages adopted different decomposition strategies. Generally, these strategies can be distinguished based on the profile of lignocellulolytic enzymes (carbohydrate-active enzymes—CAZymes) secreted by fungi during the decomposition process, and many previously published studies demonstrated a strong relationship between the CAZyme content of fungal genomes with their degradation ability [4,12,13,14,15].

Although investigation of the systematics and ecology of wood-rotting fungi has been pursued for almost two hundred years, biochemical and molecular biology aspects of fungal wood decomposition ability began to attract serious attention from researchers only in the past three decades. Recently, the *Polyporales* order was divided into four main clades: the Core Polyporoid, the Phlebioid, the Antrodia and the Residual Polyporoid [16,17]. Until now, the most considerable effort has been put into the study of fungi from the Core Polyporoid clade, predominantly genus *Trametes*, which include model wood rotting fungus *Trametes versicolor*. Today, more than 30 genomes are sequenced for the representatives of the Core Polyporoid clade (JGI database) [18], and more than 1000 articles (PubMed search) [19] have been published on different aspects of their biochemistry and molecular biology. Unfortunately, virtually all representatives of the Core Polyporoid clade can be considered as primary colonizers, making secondary colonizing fungi extremely understudied. All available information about secondary colonizing fungi is very scarce and unsystematic, which produce a considerable gap in the current knowledge of the overall wood decomposition process.

*Steccherinaceae* is a family consisting of about 200 fungal species from the Residual Polyporoid clade, many of which are secondary colonizers preferring heavily decomposed wood. Being widely distributed in forest ecosystems throughout the world, these fungi are of broad ecological interest [20,21]. Moreover, some members of this family were previously reported as fungi with high biotechnological potential [22,23,24,25,26]. *Steccherinum ochraceum* is a typical representative of the *Steccherinaceae* family. Having a wide ecological amplitude and ability to grow on a broad range of hardwood (seldomly softwood) substrates, this wood-rotting fungus can potentially be a good model for studying fungal adaptation to the ecological niche of pre-degraded wood.

In this article, we present the *de novo* assembly and annotation of the genome of *S. ochraceum*, LE-BIN 3174. This is the first published genome of the fungus from the *Steccherinaceae* family and the 9th genome sequenced for the fungus from the Residual Polyporoid clade.

Based on the sequenced genomes, the CAZymes repertoire of *S. ochraceum* LE-BIN 3174 was inferred, compared and contrasted with those of the 8 fungi belonging to the *Polyporales* and *Agaricales* orders and occupying different ecological niches and trophic groups—primary and secondary colonizer saprotrophs on *lignum*, *folia dejecta*, *stramentum*, and *humus*. Additionally, the laccases and ligninolytic peroxidases of *S. ochraceum* LE-BIN 3174 were phylogenetically positioned in the global phylogenetic trees previously constructed for these main fungal-secreted ligninolytic enzymes.

Genome-based findings regarding the intermediate position of *S. ochraceum* LE-BIN 3174 in terms of CAZymes repertoire and the peculiar evolutionary history of its laccases and ligninolytic peroxidases were further substantiated by a series of standard biochemical tests and study of the exoproteome obtained after the cultivation in the presence of wood sawdust.

## 2. Materials and Methods

### 2.1. Fungal Strains

The fungal strain of *Steccherinum ochraceum* (Persoon, 1801:Fries, 1821) Gray, 1821 was isolated (August 1, 2013) from basidiospores collected from a fallen dry aspen branch in the polydominant temperate deciduous broadleaf forest (Kaluzhskiye Zaseki Nature Reserve, Russia; N 53°33′28.4″; E 35°38′24.4″). After morphological and genetic verifications, the strain was deposited in the Komarov Botanical Institute Basidiomycetes Culture Collection (LE-BIN; St. Petersburg, Russia) as *S. ochraceum* LE-BIN 3174. The sequence of its ITS1-5.8S rRNA-ITS2 region was obtained as described in [22] and deposited into the NCBI GenBank (Table S1).

The fungal strains *Trametes versicolor* LE-BIN 2599, *Trametes pubescens* LE-BIN 3173, *Trametes hirsuta* LE-BIN 072, *Gymnopilus junonius* LE-BIN 2840, *Hymenopellis radicata* LE-BIN 1795, *Mycena galopus* LE-BIN 2249, and *Crucibulum laeve* LE-BIN 1700, *Agrocybe praecox* LE-BIN 2506 were also obtained from LE-BIN. Upon reception, genetic verification was performed for all strains, as described in [22], and the sequences of corresponding ITS1-5.8S rRNA-ITS2 regions were deposited into the NCBI GenBank (Table S1).

In the laboratory, all strains were stored on wort agar slants at 4 °C.

### 2.2. Genomic DNA Isolation, Library Preparation and Sequencing

For DNA extraction, *S. ochraceum* LE-BIN 3174 was statically cultivated at 26–28 °C in 750 mL Erlenmeyer flasks with 200 mL of glucose–peptone (GP) medium (per 1 L of dH_2_O): 3.0 g peptone, 10.0 g glucose, 0.6 g KH_2_PO_4_, 0.4 g K_2_HPO_4_, 0.5 g MgSO_4_, 50 mg MnSO_4_, 1 mg ZnSO_4_, and 0.5 mg FeSO_4_. The mycelium was ground in liquid nitrogen, and total DNA was extracted using DNeasy Plant Mini Kit (Qiagen, Valencia, CA, USA). The quality and quantity of the isolated DNA were checked using an Agilent Bioanalyzer 2100 (Agilent Technologies, Foster City, CA, USA) and Qubit fluorimeter (Thermo Fisher Scientific, Waltham, MA, USA).

After ultrasonic fragmentation, the genomic DNA was prepared for sequencing using TruSeq DNA Sample Prep Kit (Illumina, San Diego, CA, USA). The quality and quantity of the obtained DNA library was checked using Agilent Bioanalyzer 2100 and StepOnePlus Real-Time PCR System (Thermo Fisher Scientific). Whole genome sequencing was carried out with an Illumina HiSeq 2500 system (Illumina, San Diego, CA, USA) using HiSeq Rapid SBS Kit v2 at the Evrogen JSC (Moscow, Russia).

### 2.3. Genome Assembly and Annotation

The shotgun sequencing produced 2 × 47,868,586 paired-end reads (2 × 100 bp), with an insert size of 300–500 bp. The reads were further processed with CLC Genomics Workbench 11.0 (Qiagen, Valencia, CA, USA) as follows: (1) adapters were removed from all reads; (2) all reads were trimmed based on their quality; (3) reads were sampled to reduce coverage to a maximum average coverage of 100×; (4) reads were de novo assembled and resulted contigs were scaffolded.

Genome structural and functional annotations were performed using Funannotate pipeline v1.5.0 (https://github.com/nextgenusfs/funannotate).

Structural annotation step included: (1) repeat masking with the RepeatMasker package (http://www.repeatmasker.org/) using the RepBase repeats libraries [27]; (2) ab initio protein-coding gene prediction with self-trained GeneMark-ES [28] and AUGUSTUS [29], trained using BUSCO 2.0 [30] gene models (*Phanerochaete chrysosporium* was selected as a closely-related species); (3) ab initio tRNA-coding gene prediction with tRNAscan-SE [31]; (4) integration and filtering of the obtained gene models.

Functional annotation was performed with the Pfam [32], InterPro [33], eggNOG [34], dbCAN [35], MEROPS [36], antiSMASH [37], and BUSCO [30] databases. The prediction of transmembrane topologies and signal peptides was performed with Phobius [38] and SignalP [39], respectively.

### 2.4. Comparative Analysis of the CAZyme Content

For the comparative analysis of the CAZyme content, the genomes of *T. versicolor* (v1.0), *T. pubescens* (FBCC735), *G. junonius* (AH 44721 v1.0), *M. galopus* (ATCC-62051 v1.0), *C. laeve* (CBS 166.37 v1.0) and *A. praecox* (OKM6292 v1.0) were extracted from the JGI (Joint Genome Institute) portal [40]; the genomes of *T. hirsuta* (CP019370.1-CP019382.1) and *H. radicata* (QLOW00000000.1) were extracted from the GeneBank database [41]. To reduce potential bias, CAZymes content in all genomes was determined using the same pipeline as for the *S. ochraceum* LE-BIN 3174 genome, as described earlier.

### 2.5. Phylogenetic Analysis of the Laccase and Ligninolytic Peroxidase Genes

Codon-based multiple sequence alignment of the collected nucleotide sequences was constructed using the MUSCLE algorithm [42,43]. A suitable nucleotide substitution model, GTR + Γ + I, was determined using jModelTest2 software [44] under the Akaike information criterion (AIC). A phylogenetic tree was reconstructed under the maximum likelihood (ML) criterion with RAxML-HPC BlackBox (8.2.10) program [45] at the CIPRES Science Gateway [46] (https://www.phylo.org/).

### 2.6. Biochemical Tests

To obtain inoculum plugs, fungi were cultivated on MEA medium (1.5% *w*/*v* of malt extract (Conda, Spain), and 2% *w*/*v* of agar (Difco, Kansas City, MO, USA)) in Petri dishes (Ø 90 mm) at 25 °C in the dark for 10–15 days.

Express assays of oxidative enzymes were carried out in Petri dishes (Ø 90 mm) containing 20 mL of MEA and 0.1% *w*/*v* of 2,2′-azino-bis 3-ethylbenzothiazoline-6-sulfonic acid (ABTS; Sigma, St. Louis, MO, USA). Inoculum plugs (Ø 7 mm) of studied fungi were placed mycelium up on the medium and incubated at 25 °C in the dark for 48 h. After incubation, blue colored zones, which indicated the enzymatic activity, were measured.

To evaluate cellulolytic activity, mycelium plugs were incubated on a medium containing 1% *w*/*v* of carboxymethyl cellulose (CMC; Chemapol, Praha, Czech Republic) and 1 % *w*/*v* of agar (Difco, Kansas City, MO, USA) in Petri dishes (Ø 90 mm) at 25 °C. Cellulolytic activity was evaluated after 48 h by measuring decolorized zones around the inoculum. The zones were revealed using water solution of I_2_ in KI (0.5 % of I_2_ in 2 % KI), as described in [47].

Azur B decolorization assay was carried out in Petri dishes (Ø 90 mm) containing 20 mL of MEA and 75 mg/L of Azur B (Sigma, St. Louis, MO, USA). Plates with Azur B, inoculated as described above, were incubated at 25 °C in the dark and examined each 2 days for decolorization.

### 2.7. Exoproteome Study

*S. ochraceum* LE-BIN 3174 was statically cultivated for 22 days in the GP medium in the presence of lignocellulose (25 g/L of wood sawdust). The exoproteome extraction, sample preparations, two-dimensional gel electrophoresis (2-DE), MALDI-TOF/TOF MS analysis, and data processing were performed as previously described in [48].

## 3. Results and Discussion

### 3.1. Sequencing and Annotation of the S. ochraceum Genome

Using the Illumina technology, the genome of the fungus *S. ochraceum* strain LE-BIN 3174 was sequenced with overall coverage of 100× and ultimately assembled into 770 scaffolds with N50 values of 62,812 bp (the longest scaffold was 464,123 bp; mean size of scaffolds was 45,812 bp; median size of scaffolds was 33,955 bp). The final assembly comprised 35 Mb.

In the assembled genome, a total of 12,441 genes were predicted, of which 12,260 genes were identified as protein-coding, with a mean protein size of 483 aa, and 181 genes were identified as tRNA-coding. The proportion of the genome covered by the genes was 61.4%, and repeat content was 1.4%. The overall GC content of the genome was 52.7%, while coding region GC content was 54.9%.

**Figure 1:**
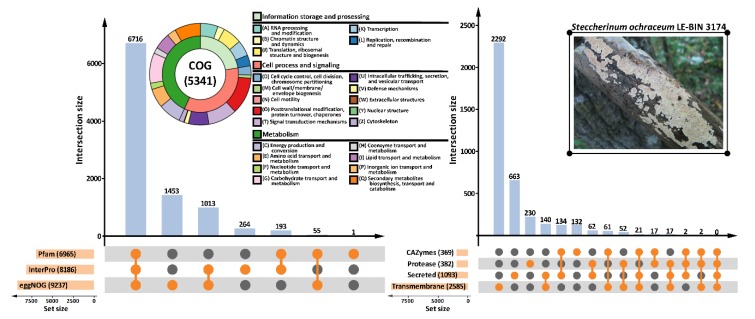
Functional annotation of the *S. ochraceum* LE-BIN 3174 genome. Number of annotations produced with different databases as well as number of gene models that received these annotations are represented with the UpSet plots [49]. Information about clusters of orthologous groups (COG) content of the genome is summarized on the double-layer donut chart. Note: CAZymes = carbohydrate-active enzymes.

Functional annotation of the predicted protein-coding genes (Figure 1) was performed with three general content databases: the protein families database Pfam [32], the integrative protein signature database InterPro [33], and the orthologous groups database eggNOG [34]. As a result of general functional prediction, 7249 genes (59%) were assigned to clusters of orthologous groups (COGs), and 5341 (44%) genes received specific COG functional categories. Additionally, two domain-specific databases were employed: carbohydrate-active enzyme (CAZyme) database dbCAN [35], and peptidase database MEROPS [36]. The prediction of transmembrane topologies and signal peptides was performed with Phobius [38] and SignalP [39], respectively.

Fungal secondary metabolite biosynthetic gene clusters were predicted with antiSMASH (fungal version) [37]. In total, 36 gene clusters were identified, among which 17 were proposed to participate in the biosynthesis of terpenes, and one was predicted as a polyketide synthase (t1pks) cluster; functions of the remaining 18 clusters were unknown.

Additionally, the genome completeness was evaluated based on the conservation of benchmarking universal single-copy orthologs (BUSCOs) [30]. The BUSCO analysis, with *Aspergillus nidulans* selected as a seed species, showed a high degree of the genome completeness. Out of the 1312 BUSCO groups searched, 1198 genes (91.3%) were complete BUSCOs, of which 14 genes were complete duplicated BUSCOs; 76 genes (5.8%) were fragmented BUSCOs; and 38 genes (2.9%) were missing BUSCOs.

In summary, the obtained assembly and annotation of the genome of *S. ochraceum* strain LE-BIN 3174 is of comparable quality with previously published genomes of other polypore fungi [40]. Moreover, it is the first published genome of the fungus from the *Steccherinaceae* family.

The Whole Genome Shotgun project had been deposited at DDBJ/ENA/GenBank under the accession RWJN00000000. The version described in this paper is version RWJN00000000.1.

Although, as it is widely acknowledged, each new sequenced genome provides an enormous amount of information, in this article we primarily focus on the peculiarities of the *S. ochraceum* CAZymes repertoire in comparison with other fungi from different ecological niches and trophic groups.

### 3.2. The Peculiarities of the S. ochraceum CAZyme Genome Content

The whole genome sequence of *S. ochraceum* LE-BIN 3174 showed that it harbors 361 CAZymes (Table S2). The auxiliary activity enzymes (AA), carbohydrate esterase (CE), glycoside hydrolases (GH), glycosyl transferase (GT), and polysaccharide lyase (PL) superfamilies were represented by 109, 37, 151, 55, and 9 CAZymes from 9, 8, 48, 25, and 3 families, respectively. Based on the previous literature [13], identified CAZymes were classified into seven groups according to the lignocellulose’s polymeric components, upon which they can potentially act (Table S2). In total, 19 CAZymes involved in lignin degradation (8 laccases and 11 class II peroxidases) and 91 CAZymes involved in polysaccharide degradation were identified. Among polysaccharide-degrading CAZymes, 54 were related to the degradation of cellulose, 47 to hemicellulose (14–xylan, 23–galactomannan, 11–xyloglucan, and 11–arabinoxylan), and 36–pectin (please note that the numbers do not add up properly due to the redundancy in the classification scheme, which was advanced to reflect different enzymatic activities possessed by fungi rather than different CAZymes, since the same CAZyme can simultaneously act on several components of lignocellulose) (Table S2).

**Figure 2:**
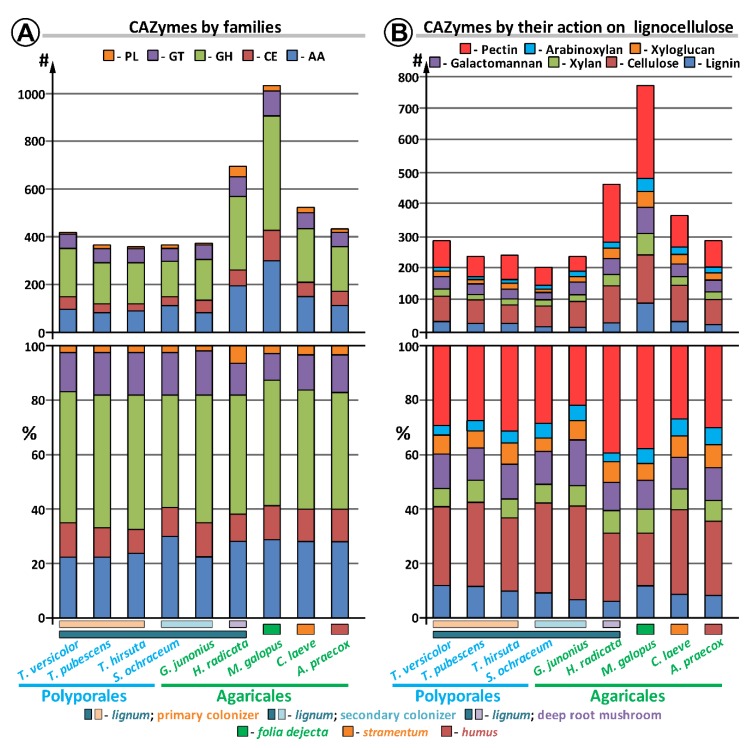
Comparison of the CAZyme repertoire in *S. ochraceum* and other fungal genomes.

Comparison of the *S. ochraceum* CAZyme genome content with those from other lignocellulose decaying fungi belonging to the different trophic groups (Figure 2, Table S1) revealed its several peculiarities.

For the total CAZyme content (Figure 2, Table S2), the comparison demonstrated that the number of CAZymes and their group-wise distribution in *S. ochraceum* are generally similar to those of the primary colonizing fungi (i.e., *T. versicolor*, *T. pubescens*, *T. hirsuta*). The most discrepancies for these fungi were observed for the GH and AA superfamilies. In the GH superfamily, the number of CAZymes was reduced (151 vs. average of 183), while in the AA superfamily the number was elevated (109 vs. average of 87). For the GH superfamily, the total difference in the number of CAZymes was almost uniformly spread among all its families. For the AA superfamily, the number of CAZymes from the AA3, AA6, and AA7 families was greatly elevated (44 vs. average of 25 for AA3; 4 vs. average of 1 for AA6; and 16 vs. averages of 5 for AA7), while the number of CAZymes from the AA2 family was significantly reduced (11 vs. average of 20). Additionally, slight elevation in the number of CAZymes from the AA1_1 family (8 vs. averages of 6) should be noted. Hence, for these five AA families, *S. ochraceum* is closer to the litter decomposers and humus saprotrophs. Moreover, since the AA7 family contains gluco-oligosaccharide oxidases (EC 1.1.3.-) that are involved in degradation of partially hydrolyzed cellulose biopolymer, and as the AA6 and AA1_1 families contain 1,4-benzoquinone reductases (EC 1.6.5.6) and laccases (EC 1.10.3.2) that are involved in the detoxification of different aromatic compounds that can be formed after lignin depolymerization, expansion of these families can be seen as a reflection of the substrate preferences of *S. ochraceum* toward partially degraded wood. Similarly, the reduction of the AA2 family containing fungal class II ligninolytic peroxidases (EC 1.11.1.-) that actively participate in the lignin oxidative depolymerization reflects decreased demand for such a process by the secondary colonizer fungus.

Considering the CAZymes acting on different polymeric components of lignocellulose [13], among all fungi, *S. ochraceum* generally possesses the smallest number of CAZymes in each group under consideration (Figure 2, Table S3). This again could reflect the preference of this fungus to the partially degraded wood that contains less complex polymeric components.

### 3.3. The Evolutionary History of S. ochraceum Laccase and Ligninolytic Peroxidase Genes

It is fair to say that among all CAZymes of wood-degrading fungi, laccases and ligninolytic peroxidases (i.e., manganese peroxidases (MnPs), lignin peroxidases (LiPs), and versatile peroxidases (VPs)) are the enzymes that currently attract the most attention from researchers [50,51,52]. Despite the long history of investigation (the first fungal laccase was discovered in 1896 [53,54], and the first ligninolytic peroxidase in 1983 [55]), it is only recently that the research community has started to fully appreciate the natural variability of these enzymes. As is shown for more than 300 genomes of Basidiomycete fungi, both laccases and ligninolytic peroxidases always form multigene families [40]. So, each fungus can potentially produce many laccase and peroxidase isozymes (i.e., products of different nonallelic genes), each of which can be presented by several isoforms (different in posttranslational modifications, e.g., glycosylation). Currently, since different biotechnological processes become more and more demanding for the specific properties of utilized enzymes (e.g., thermostability and substrate specificity), natural variability of laccases and peroxidases starts to stimulate their deeper investigation, and more importantly, systematization [56,57].

The genome of *S. ochraceum* strain LE-BIN 3174 contains 8 laccase and 11 peroxidase genes. In the current report, the determined genes were positioned in the existing phylogenetic framework, which for laccases was previously reported in [58] and for peroxides in [59].

**Figure 3:**
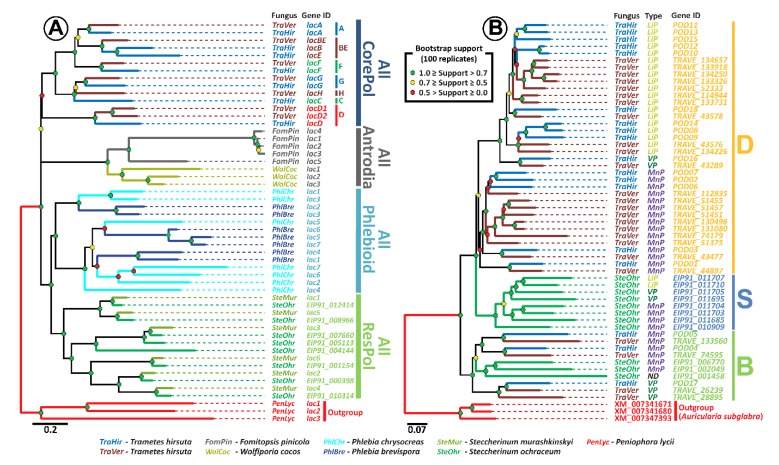
(**A**) Maximum likelihood (ML) phylogenetic tree of laccase genes (tree annotation is consistent with those from more general tree in [58]). (**B**) ML phylogenetic tree of peroxidase genes (tree annotation is consistent with those from more general tree in [59]).

In case of the laccases (Figure 3A), the constructed phylogenetic tree gives additional support to the hypothesis previously proposed in [58], stating that laccases of the fungi from the Residual Polyporoid clade form an evolutionary distinct group (all ResPol subclade). Hence, the expansion of the laccase multigene family in the genomes of these fungi proceeded independently (in parallel) from its expansion in the genomes of fungi from the other main clade of the *Polyporales* order, in particular from the fungi of the evolutionally closest Phlebioid clade, which is contrary to the possible inheritance of multiple laccase genes from the common for these clades’ ancestor fungal population.

Additionally, the strong pattern of pairing of the laccase genes of *S. ochraceum* with the laccase genes of *Steccherinum murashkinskyi* on our tree suggests that for the fungi from the *Steccherinaceae* family, multiple laccase genes were probably inherited from its common ancestral population. The similar situation was previously described for the fungi from the Core Polyporoid clade [58]. For this clade, multiple duplications of the original laccase gene were predicted in its last common ancestor fungal population, and the approximate time of these duplications was determined to be near the second half of the early Cretaceous, the time when angiosperm plants were undergoing major radiation that produced a new ecological niche for the wood-rotting fungi [60,61,62]. Interestingly, the previously conducted in [62,63,64] molecular clock analysis suggests that the ancestral population that gave rise to the fungi of the *Steccherinaceae* family was formed near the end of the early Cretaceous; hence, multiple laccase duplications in this population can be promoted by the same necessity for adaptation to the wood of angiosperm plants.

Phylogenetic analysis of the *S. ochraceum* ligninolytic peroxidase genes and its comparison with previously published data [59] suggest the presence of two groups of peroxidases in this fungus (Figure 3B). The first group is positioned within clade B, previously described in [59]. This clade contains peroxidases, which ancestral genes duplicated and diversified into VPs and MnPs before splitting of the *Polyporales* order into its four main clades at the end of the Jurassic [62,63,64]. The second group forms clade S, which is newly identified in this study. Since this clade solely contains the peroxidases of *S. ochraceum*, it can be concluded that the ancestral for this clade peroxidase gene started to duplicate after the formation of the evolutionary branch that leads to the *Steccherinaceae* family.

More detailed analysis revealed that the relationships between different types of peroxidases in clade S resemble those in clade D. Clade D was also described in [59], and contains peroxidases of the fungi from the Core Polyporoid clade, whose ancestral gene duplicated and diversified into MnPs, VPs, and LiPs during the Cretaceous [63]. Resemblance of the phylogenetic relationships between clade S and clade D suggested that duplicated genes in clade S underwent the same sequence of diversification that was previously described for clade D [59]: (1) incorporation of an exposed tryptophan residue by one of the ancient MnP that became a first VP; (2) duplication(s) of ancient VP gene; (3) loss of the Mn(II)-oxidation site by one of the ancient VPs that gave rise to all LiPs. In contrast to clade D, clade S can be characterized by the smaller number of duplications of LiPs. Interestingly, in clade D, a major number of duplications of LiPs occurred at the levels of individual species, all of which are primary colonizers. Hence, it can be speculated that when *S. ochraceum* adopted a secondary colonization strategy, expansion of its LiP stopped.

### 3.4. Biochemical Plate Tests

To substantiate our findings about the peculiarities of *S. ochraceum* LE-BIN 3174 CAZyme genome content, additional series of comparative plate tests were performed (Figure S1). In these tests, two substrates (ABTS and Azur B) were used to assess general oxidative ability of fungal mycelia. While ABTS is regarded as a readily degradable substrate that can be potentially oxidized by a variety of enzymes (e.g., laccases and ligninolytic peroxidases), Azur B can be degraded only by high-redox-potential enzymes, such as ligninolytic peroxidases. To assess the general cellulolytic activity of fungal mycelia toward microcrystalline cellulose, CMC was used as a substrate. For comparative purposes, the pure cultures of the 9 fungal species previously selected for the comparison of the CAZymes genome contents were used (Table S1).

**Figure 4:**
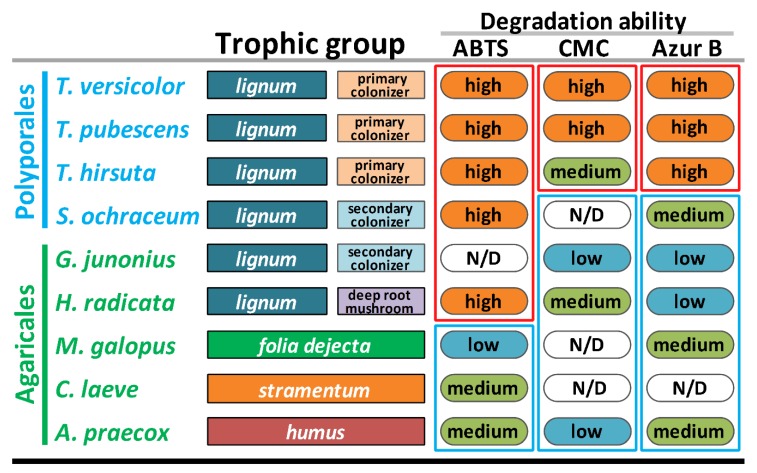
Ecological and biochemical characteristics of the selected fungi. Note: ABTS = 2,2′-azino-bis 3-ethylbenzothiazoline-6-sulfonic acid; CMC = carboxymethyl cellulose.

As can be seen in Figure 4, *S. ochraceum*, demonstrating high degradation ability toward ABTS, forms a group with all fungi (except *G. junonius*) that have *lignum* as a primary substrate. In contrast, medium degradation ability toward Azur B placed *S. ochraceum* in the group with the litter decomposers and humus saprotrophs, apart from the primary colonizers.

In general, the medium ability of *S. ochraceum* to degrade Azur B suggests that its extracellular enzymes have a low capability to degrade non-phenolic lignin structures, and thus, actively depolymerize lignin. At the same time, high degradation ability toward ABTS proposed the presence of an enzymatic system that can potentially oxidize phenolic structures of lignin, and in doing this facilitate their polymerization and detoxification, which is a necessary process during the occupation of a pre-degraded wood.

It is interesting that the main extracellular enzymes responsible for the degradation of both ABTS and Azur B belong to the AA CAZyme superfamily—by the genome content of which *S. ochraceum* was demonstrated to be closer to the litter decomposers and humus saprotrophs rather than *lignocolus* (wood-inhabiting) fungi. Hence, the intermediate position of *S. ochraceum* between these two groups of fungi, predicted based on the AA-CAZyme genome content, was clearly substantiated at the level of its overall biochemistry.

For the degradation ability toward CMC, *S. ochraceum* groups together with the litter decomposers and humus saprotrophs, the degradation ability of which was either low or undetected. Again, these data correlate with the smaller number of GH-CAZymes in the genome of *S. ochraceum* and support the proposition that as a secondary colonizer this fungus does not need an extensive enzyme complex to attack crystalline cellulose.

### 3.5. The Exoproteome of S. ochraceum Cultivated on the Lignocellulose Substrate

**Figure 5:**
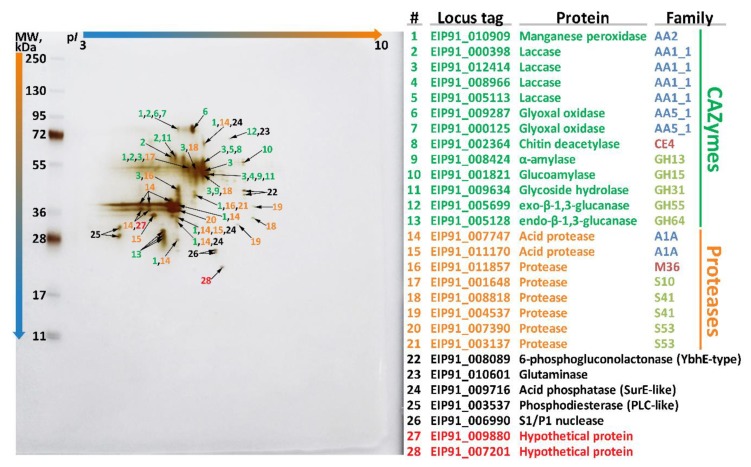
Two-dimensional gel electrophoresis (2DE) of the exoproteome of *S. ochraceum* cultivated on the lignocellulose substrate.

To go deeper at the molecular level, an additional exoproteome study was performed. During this study, the exoproteome of *S. ochraceum* cultivated in the presence of wood sawdust was extracted and analyzed using two-dimensional gel electrophoresis followed by mass spectrometry (MS). Using the unique peptides detected by MS and the performed structural annotation of the genome, 28 distinct proteins in the exoproteome were identified (Figure 5). At the same time, the performed functional genome annotation allowed assigning specific enzymatic activities to all but two identified proteins.

The major part of the identified proteins (13) belonged to the CAZyme functional group, among which the AA superfamily was represented by 7 enzymes: one manganese peroxidase, four distinct laccases, and two distinct glyoxal oxidases (EC 1.2.3.15); the CE superfamily was represented by one enzyme: chitin deacetylase (EC 3.5.1.41); and the GH superfamily was represented by 5 enzymes: alpha-amylase (EC 3.2.1.1), glucoamylase (EC 3.2.1.3), glycoside hydrolase (EC 3.2.1.-), exo-beta-1,3-glucanase (EC 3.2.1.58), and endo-beta-1,3-glucanase (EC 3.2.1.39).

Regarding the main ligninolytic CAZymes, in contrast with the previously published exoproteome of *T. hirsuta* [48] that was obtained under the similar conditions, the exoproteome of *S. ochraceum* (Figure 5) was characterized by a broader spectrum of laccase isozymes (four vs. one) and narrower spectrum of peroxidase isozymes (one vs. three). Additionally, the only peroxidase secreted by *S. ochraceum* (EIP91_010909) belonged to the evolutionary distinct clade S (Figure 3), and its secreted amount was significantly lower than those of laccases. This difference in the exoproteomes of these two fungi presumably illustrates a general tendency—for the fungi participating in the early stages of wood degradation it is advantageous to produce more peroxidases (both in number of isozymes and amount of secreted proteins) than laccases, while for the fungi participating in the later stages of wood degradation, exactly the opposite is true. This proposition can be partly supported by the fact that for other secondary colonizing fungi from the *Steccherinaceae* family (*Steccherinum bourdotii* and *Junghuhnia nitida*), prevalence of laccase activity over peroxidase was previously demonstrated during the growth on a lignocellulose substrate [22]. On the contrary, for many primary colonizing fungi (mainly from the *Trametaceae* family), the prevalence of peroxidases over laccase in exoproteome is well documented [65,66].

As we have previously shown, the substrate specificity and the structure of loops near the substrate binding pockets of laccases from the fungi of the *Steccherinaceae* and *Trametaceae* families differ significantly [22,23]. What is especially interesting is that the substrate specificity of the laccases from the fungi of the *Steccherinaceae* family, including *S. ochraceum*, are more similar to the substrate specificity of laccases of the litter and humus saprotrophs [22,23]. Moreover, many litter and humus saprotrophs demonstrate high laccase and low peroxidase activity during their growth on lignocellulose substrates [67,68,69]. Hence, both secondary colonizers and litter and humus saprotrophs evolved—most probably in parallel—laccases with similar substrate specificities, which they prefer to use over their peroxidases.

Regarding the cellulolytic CAZymes, the obtained exoproteome of *S. ochraceum* (Figure 5) was characterized by virtual absence of the CAZymes specifically devoted to the degradation of crystalline cellulose. This is in line with the reduced number of corresponding CAZymes in the *S. ochraceum* genome (especially family GH5, which include β-1,4-endoglucanase), and the undetectable by the plate tests cellulolytic activity. In contrast, exoproteomes of the white rot fungi that perform early phase of wood degradation are typically characterized by the presence of a full spectrum of glycoside hydrolases, which are involved in degradation of cell wall cellulose, hemicelluloses, and pectin (Table S3) [48,70].

The second largest group of enzymes detected in the exoproteome of *S. ochraceum* (Figure 5) consisted of different proteases belonging to the 5 distinct protease families [36]: two acid proteases from A1A family; 5 serine proteases—two from S53, two from S41 and one from S10 family; and one metalloprotease from M56 family. Secretion of many proteases that are diverse in nature by *S. ochraceum* could be due to the fact that for the secondary colonizing fungi, mycelia of the fungi from the earlier successional stages can serve as a direct source of nutrition [71]. Interestingly, the described situation is very common for litter and humus saprotrophs, many of which can use proteins as a sole source of carbon and nitrogen, and consequently highly express a variety of different proteases [72]. In contrast, primary colonizing fungi typically do not extensively produce proteases; for example, in the exoproteome of *T. hirsuta*, just one protease was detected [48].

The remaining part of the exoproteome of *S. ochraceum* (Figure 5) is represented by the different enzymes that increase nutrient acquisition functions: 6-phosphogluconolactonase (EC 3.1.1.31), glutaminase (EC 3.5.1.2), acid phosphatase (EC 3.1.3.2), phosphodiesterase (EC 3.1.4.-), and S1/P1 nuclease (EC 3.1.30.1). It is well known that production of such enzymes along with different proteases is an important hallmark of fungi that frequently participate in antagonistic interactions with their neighbors [71,73]. With an advancement of degradation, the fungal biodiversity in wood together with the number of antagonistic interactions between different fungal strains increases [74]; as such, it is expected for the secondary colonizing fungi to possess an array of enzymes that allow them to reuse remnants of both their dead predecessors and neighbors.

## 4. Conclusions

The genome of *S. ochraceum* announced in this article, strain LE-BIN 3174, together with the data from the biochemical plate tests and exoproteomic study allowed us to gain the first glimpse into the fungal adaptation to the advanced stages of wood decomposition.

With the first sequenced genome of the fungus from the *Steccherinaceae* family, we had the unique opportunity to explore the evolution of the two main secreted fungal oxidoreductases—laccases and class II peroxidases. We demonstrated that *S. ochraceum* and probably all fungi from the *Steccherinaceae* family possess a distinct phylogenetic group of these enzymes that started to form after the *Steccherinaceae* branch budded off near the end of the early Cretaceous.

The performed comparative analysis of the CAZyme genome content suggested that as a secondary colonizer, *S. ochraceum* possess more CAZymes belonging to the AA superfamily and less CAZymes belonging to the GH superfamily, in contrast to the primary colonizing fungi. Moreover, for several CAZymes from the AA superfamily (i.e., AA1_1, AA2, AA3, AA6, and AA7), *S. ochraceum* assumes the intermediate position between typical primary colonizing fungi and litter decomposers or humus saprotrophs. Importantly, the described intermediate position of *S. ochraceum* was subsequently confirmed both by the plate tests and exoproteomic study. The plate tests demonstrated that, possessing high degradation ability toward ABTS, *S. ochraceum* groups together with litter decomposers and humus saprotrophs, while, possessing low degradation ability toward Azur B and undetectable—toward CMC, *S. ochraceum* groups with primary colonizing fungi. Exoproteomic study revealed that the most abundant protein category secreted by *S. ochraceum* in the presence of wood sawdust is oxidoreductases (mainly laccases, and proteases), which is more typical for litter decomposers and humus saprotrophs than for primary colonizing fungi.

## Supplementary Materials

The following are available online at https://www.mdpi.com/2076-2607/7/11/527/s1. Figure S1: Oxidation activity (ABTS), cellulolytic activity (CMC), and Azur B decolorization (Azur B) by different fungi. Table S1: The fungal strains. Table S2: Carbohydrate-degrading enzymes in *Steccherinum ochraceum* LE-BIN 3174 and other basidiomycetes. Table S3: Comparative analysis of the number of CAZymes families related to plant polysaccharide degradation in *Steccherinum ochraceum* LE-BIN 3174 and other basidiomycetes.

## References

[1] Nabuurs G.J., Päivinen R., Sikkema R., Mohren G.M.J. (1997). The role of European forests in the global carbon cycle-a review. Biomass Bioenergy.

[2] Köhl M., Lasco R., Cifuentes M., Jonsson Ö., Korhonen K.T., Mundhenk P., de Jesus Navar J., Stinson G. (2015). Changes in forest production, biomass and carbon: Results from the 2015 UN FAO global forest resource assessment. For. Ecol. Manag..

[3] Cragg S.M., Beckham G.T., Bruce N.C., Bugg T.D.H., Distel D.L., Dupree P., Etxabe A.G., Goodell B.S., Jellison J., McGeehan J.E. (2015). Lignocellulose degradation mechanisms across the tree of life. Curr. Opin. Chem. Biol..

[4] Lundell T.K., Mäkelä M.R., de Vries R.P., Hildén K.S. (2014). Genomics, lifestyles and future prospects of wood-decay and litter-decomposing Basidiomycota. Adv. Bot. Res..

[5] Ismail-Meyer K., Stolt M.H., Lindbo D.L. (2018). Soil organic matter. Interpret. Micromorphol. Features Soils Regoliths.

[6] Waring R.H., Running S.W. (2007). Mineral cycles. For. Ecosyst..

[7] Lonsdale D., Pautasso M., Holdenrieder O. (2008). Wood-decaying fungi in the forest: Conservation needs and management options. Eur. J. For. Res..

[8] Marcot B.G. (2017). A review of the role of fungi in wood decay of forest ecosystems. US Dep. Agric. For. Serv. Pac. Northwest Res. Stn..

[9] Jönsson M.T., Edman M., Jonsson B.G. (2008). Colonization and extinction patterns of wood-decaying fungi in a boreal old-growth *Picea abies* forest. J. Ecol..

[10] Boddy L., Frankland J., van West P. (2008). Ecology of Saprotrophic Basidiomycetes.

[11] Boddy L. (2001). Fungal community ecology and wood decomposition processes in Angiosperms: From standing tree to complete decay of coarse woody debris. Ecol. Bull..

[12] Riley R., Salamov A.A., Brown D.W., Nagy L.G., Floudas D., Held B.W., Levasseur A., Lombard V., Morin E., Otillar R. (2014). Extensive sampling of basidiomycete genomes demonstrates inadequacy of the white-rot/brown-rot paradigm for wood decay fungi. Proc. Natl. Acad. Sci. USA.

[13] Rytioja J., Hildén K., Yuzon J., Hatakka A., de Vries R.P., Mäkelä M.R. (2014). Plant-polysaccharide-degrading enzymes from Basidiomycetes. Microbiol. Mol. Biol. Rev..

[14] Sista Kameshwar A.K., Qin W. (2018). Comparative study of genome-wide plant biomass-degrading CAZymes in white rot, brown rot and soft rot fungi. Mycology.

[15] Hori C., Gaskell J., Igarashi K., Samejima M., Hibbett D., Henrissat B., Cullen D. (2013). Genomewide analysis of polysaccharides degrading enzymes in 11 white- and brown-rot Polyporales provides insight into mechanisms of wood decay. Mycologia.

[16] Binder M., Justo A., Riley R., Salamov A., Lopez-Giraldez F., Sjokvist E., Copeland A., Foster B., Sun H., Larsson E. (2013). Phylogenetic and phylogenomic overview of the Polyporales. Mycologia.

[17] Justo A., Miettinen O., Floudas D., Ortiz-Santana B., Sjökvist E., Lindner D., Nakasone K., Niemelä T., Larsson K.-H., Ryvarden L. (2017). A revised family-level classification of the Polyporales (*Basidiomycota*). Fungal Biol..

[18] Grigoriev I.V., Nikitin R., Haridas S., Kuo A., Ohm R., Otillar R., Riley R., Salamov A., Zhao X., Korzeniewski F. (2013). MycoCosm portal: Gearing up for 1000 fungal genomes. Nucleic Acids Res..

[19] Agarwala R., Barrett T., Beck J., Benson D.A., Bollin C., Bolton E., Bourexis D., Brister J.R., Bryant S.H., Canese K. (2018). Database resources of the National Center for Biotechnology Information. Nucleic Acids Res..

[20] Miettinen O., Ryvarden L. (2016). Polypore genera *Antella*, *Austeria*, *Butyrea*, *Citripora*, *Metuloidea* and *Trulla* (*Steccherinaceae*, Polyporales). Ann. Bot. Fenn..

[21] Fedorova T.V., Shakhova N.V., Klein O.I., Glazunova O.A., Maloshenok L.G., Kulikova N.A., Psurtseva N.V., Koroleva O.V. (2013). Comparative analysis of the ligninolytic potential of basidiomycetes belonging to different taxonomic and ecological groups. Appl. Biochem. Microbiol..

[22] Glazunova O.A., Shakhova N.V., Psurtseva N.V., Moiseenko K.V., Kleimenov S.Y., Fedorova T.V. (2018). White-rot basidiomycetes *Junghuhnia nitida* and *Steccherinum bourdotii*: Oxidative potential and laccase properties in comparison with *Trametes hirsuta* and *Coriolopsis caperata*. PLoS ONE.

[23] Glazunova O.A., Polyakov K.M., Moiseenko K.V., Kurzeev S.A., Fedorova T.V. (2018). Structure-function study of two new middle-redox potential laccases from basidiomycetes *Antrodiella faginea* and *Steccherinum murashkinskyi*. Int. J. Biol. Macromol..

[24] Myasoedova N.M., Chernykh A.M., Psurtseva N.V., Belova N.V., Golovleva L.A. (2008). New efficient producers of fungal laccases. Appl. Biochem. Microbiol..

[25] Chernykh A.M., Myasoedova N.M., Kolomytseva M., Ferraroni M., Briganti F., Scozzafava A., Golovleva L. (2008). Laccase isoforms with unusual properties from the basidiomycete *Steccherinum ochraceum* strain 1833. J. Appl. Microbiol..

[26] Glazunova O., Trushkin N., Moiseenko K., Filimonov I., Fedorova T. (2018). Catalytic efficiency of basidiomycete laccases: RedOx potential versus substrate-binding pocket structure. Catalysts.

[27] Bao W., Kojima K.K., Kohany O. (2015). Repbase Update, a database of repetitive elements in eukaryotic genomes. Mob. DNA.

[28] Besemer J., Lomsadze A., Borodovsky M. (2001). GeneMarkS: A self-training method for prediction of gene starts in microbial genomes. Implications for finding sequence motifs in regulatory regions. Nucleic Acids Res..

[29] Stanke M., Steinkamp R., Waack S., Morgenstern B. (2004). AUGUSTUS: A web server for gene finding in eukaryotes. Nucleic Acids Res..

[30] Simão F.A., Waterhouse R.M., Ioannidis P., Kriventseva E.V., Zdobnov E.M. (2015). BUSCO: Assessing genome assembly and annotation completeness with single-copy orthologs. Bioinformatics.

[31] Lowe T.M., Eddy S.R. (1997). tRNAscan-SE: A program for improved detection of transfer RNA genes in genomic sequence. Nucleic Acids Res..

[32] Finn R.D., Coggill P., Eberhardt R.Y., Eddy S.R., Mistry J., Mitchell A.L., Potter S.C., Punta M., Qureshi M., Sangrador-Vegas A. (2016). The Pfam protein families database: Towards a more sustainable future. Nucleic Acids Res..

[33] Hunter S., Apweiler R., Attwood T.K., Bairoch A., Bateman A., Binns D., Bork P., Das U., Daugherty L., Duquenne L. (2009). InterPro: The integrative protein signature database. Nucleic Acids Res..

[34] Huerta-Cepas J., Szklarczyk D., Forslund K., Cook H., Heller D., Walter M.C., Rattei T., Mende D.R., Sunagawa S., Kuhn M. (2016). eggNOG 4.5: A hierarchical orthology framework with improved functional annotations for eukaryotic, prokaryotic and viral sequences. Nucleic Acids Res..

[35] Yin Y., Mao X., Yang J., Chen X., Mao F., Xu Y. (2012). dbCAN: A web resource for automated carbohydrate-active enzyme annotation. Nucleic Acids Res..

[36] Rawlings N.D., Barrett A.J., Bateman A. (2012). MEROPS: The database of proteolytic enzymes, their substrates and inhibitors. Nucleic Acids Res..

[37] Weber T., Blin K., Duddela S., Krug D., Kim H.U., Bruccoleri R., Lee S.Y., Fischbach M.A., Müller R., Wohlleben W. (2015). antiSMASH 3.0-a comprehensive resource for the genome mining of biosynthetic gene clusters. Nucleic Acids Res..

[38] Käll L., Krogh A., Sonnhammer E.L.L. (2007). Advantages of combined transmembrane topology and signal peptide prediction-the Phobius web server. Nucleic Acids Res..

[39] Nielsen H. (2017). Predicting Secretory Proteins with SignalP.

[40] Nordberg H., Cantor M., Dusheyko S., Hua S., Poliakov A., Shabalov I., Smirnova T., Grigoriev I.V., Dubchak I. (2014). The genome portal of the Department of Energy Joint Genome Institute: 2014 updates. Nucleic Acids Res..

[41] Clark K., Karsch-Mizrachi I., Lipman D.J., Ostell J., Sayers E.W. (2016). GenBank. Nucleic Acids Res..

[42] Larsson A. (2014). AliView: A fast and lightweight alignment viewer and editor for large datasets. Bioinformatics.

[43] Edgar R.C. (2004). MUSCLE: Multiple sequence alignment with high accuracy and high throughput. Nucleic Acids Res..

[44] Darriba D., Taboada G.L., Doallo R., Posada D. (2012). jModelTest 2: More models, new heuristics and parallel computing. Nat. Methods.

[45] Stamatakis A. (2014). RAxML version 8: A tool for phylogenetic analysis and post-analysis of large phylogenies. Bioinformatics.

[46] Miller M.A., Pfeiffer W., Schwartz T. Creating the CIPRES Science Gateway for inference of large phylogenetic trees. Proceedings of the IEEE 2010 Gateway Computing Environments Workshop (GCE).

[47] Klán J., Baudisova D. (1990). Enzyme activity of mycelial cultures of saprotrophic macromycetes (*Basidiomycotina*). I. Methods of hydrolase estimation. Česká Mycol..

[48] Vasina D.V., Pavlov A.R., Koroleva O.V. (2016). Extracellular proteins of *Trametes hirsuta* st. 072 induced by copper ions and a lignocellulose substrate. BMC Microbiol..

[49] Lex A., Gehlenborg N., Strobelt H., Vuillemot R., Pfister H. (2014). UpSet: Visualization of intersecting sets. IEEE Trans. Vis. Comput. Graph..

[50] Yang J., Li W., Ng T.B., Deng X., Lin J., Ye X. (2017). Laccases: Production, expression regulation, and applications in pharmaceutical biodegradation. Front. Microbiol..

[51] Hammel K., Cullen D. (2008). Role of fungal peroxidases in biological ligninolysis. Curr. Opin. Plant Biol..

[52] Falade A.O., Nwodo U.U., Iweriebor B.C., Green E., Mabinya L.V., Okoh A.I. (2017). Lignin peroxidase functionalities and prospective applications. Microbiologyopen.

[53] Bertrand G. (1896). Sur la presence simultanee de la laccase et de la tyrosinase dans le suc de quelques champignons. C. R. Hebd. Seances Acad. Sci..

[54] Laborde J. (1896). Sur la casse des vins. C. R. Hebd. Seances Acad. Sci..

[55] Tien M., Kirk T.K. (1983). Lignin-degrading enzyme from the hymenomycete *Phanerochaete chrysosporium* Burds. Science.

[56] Sirim D., Wagner F., Wang L., Schmid R.D., Pleiss J. (2011). The Laccase Engineering Database: A classification and analysis system for laccases and related multicopper oxidases. Database.

[57] Savelli B., Li Q., Webber M., Jemmat A.M., Robitaille A., Zamocky M., Mathé C., Dunand C. (2019). RedoxiBase: A database for ROS homeostasis regulated proteins. Redox Biol..

[58] Savinova O.S., Tyazhelova T.V., Moiseenko K.V., Chulkin A.M., Vasina D.V., Vavilova E.A., Fedorova T.V. (2019). Evolutionary relationships between the laccase genes of polyporales: Orthology-based classification of laccase isozymes and functional insight from *Trametes hirsuta*. Front. Microbiol..

[59] Ruiz-Dueñas F.J., Lundell T., Floudas D., Nagy L.G., Barrasa J.M., Hibbett D.S., Martínez A.T. (2013). Lignin-degrading peroxidases in Polyporales: An evolutionary survey based on 10 sequenced genomes. Mycologia.

[60] Coiffard C., Gomez B., Daviero-Gomez V., Dilcher D.L. (2012). Rise to dominance of angiosperm pioneers in European Cretaceous environments. Proc. Natl. Acad. Sci. USA.

[61] Lupia R., Lidgard S., Crane P.R. (1999). Comparing palynological abundance and diversity: Implications for biotic replacement during the Cretaceous angiosperm radiation. Paleobiology.

[62] Krah F.-S., Bässler C., Heibl C., Soghigian J., Schaefer H., Hibbett D.S. (2018). Evolutionary dynamics of host specialization in wood-decay fungi. BMC Evol. Biol..

[63] Floudas D., Binder M., Riley R., Barry K. (2012). The Paleozoic origin of enzymatic lignin decomposition reconstructed from 31 fungal genomes. Science.

[64] Garcia-Sandoval R., Wang Z., Binder M., Hibbett D.S. (2011). Molecular phylogenetics of the Gloeophyllales and relative ages of clades of Agaricomycotina producing a brown rot. Mycologia.

[65] Presley G.N., Panisko E., Purvine S.O., Schilling J.S. (2018). Coupling secretomics with enzyme activities to compare the temporal processes of wood metabolism among white and brown rot fungi. Appl. Environ. Microbiol..

[66] Carabajal M., Kellner H., Levin L., Jehmlich N., Hofrichter M., Ullrich R. (2013). The secretome of *Trametes versicolor* grown on tomato juice medium and purification of the secreted oxidoreductases including a versatile peroxidase. J. Biotechnol..

[67] Hoppe B., Purahong W., Wubet T., Kahl T., Bauhus J., Arnstadt T., Hofrichter M., Buscot F., Krüger D. (2016). Linking molecular deadwood-inhabiting fungal diversity and community dynamics to ecosystem functions and processes in Central European forests. Fungal Divers..

[68] Liers C., Arnstadt T., Ullrich R., Hofrichter M. (2011). Patterns of lignin degradation and oxidative enzyme secretion by different wood- and litter-colonizing basidiomycetes and ascomycetes grown on beech-wood. FEMS Microbiol. Ecol..

[69] Gupta D.K., Rühl M., Mishra B., Kleofas V., Hofrichter M., Herzog R., Pecyna M.J., Sharma R., Kellner H., Hennicke F. (2018). The genome sequence of the commercially cultivated mushroom *Agrocybe aegerita* reveals a conserved repertoire of fruiting-related genes and a versatile suite of biopolymer-degrading enzymes. BMC Genom..

[70] Rytioja J., Hildén K., Di Falco M., Zhou M., Aguilar-Pontes M.V., Sietiö O.-M., Tsang A., de Vries R.P., Mäkelä M.R. (2017). The molecular response of the white-rot fungus *Dichomitus squalens* to wood and non-woody biomass as examined by transcriptome and exoproteome analyses. Environ. Microbiol..

[71] Hiscox J., Boddy L. (2017). Armed and dangerous-chemical warfare in wood decay communities. Fungal Biol. Rev..

[72] Morin E., Kohler A., Baker A.R., Foulongne-Oriol M., Lombard V., Nagye L.G., Ohm R.A., Patyshakuliyeva A., Brun A., Aerts A.L. (2012). Genome sequence of the button mushroom *Agaricus bisporus* reveals mechanisms governing adaptation to a humic-rich ecological niche. Proc. Natl. Acad. Sci. USA.

[73] Lindahl B.D., Finlay R.D. (2006). Activities of chitinolytic enzymes during primary and secondary colonization of wood by basidiomycetous fungi. New Phytol..

[74] Mäkipää R., Rajala T., Schigel D., Rinne K.T., Pennanen T., Abrego N., Ovaskainen O. (2017). Interactions between soil- and dead wood-inhabiting fungal communities during the decay of Norway spruce logs. ISME J..

